# Poor glycemic control and associated factors in diabetic people attending a reference outpatient clinic in Mato Grosso, Brazil

**DOI:** 10.17533/udea.iee.v39n3e10

**Published:** 2021-11-08

**Authors:** Mariano Martínez Espinosa, Vitesinha Rosa dos Santos Almeida, Vagner Ferreira do Nascimento

**Affiliations:** 1 Statistician, Ph.D. Professor at the Department of Statistics Universidade Federal de Mato Grosso (UFMT) Cuiabá - MT Brazil. Email: marianomphd@gmail.com Universidade Federal de Mato Grosso Department of Statistics Universidade Federal de Mato Grosso Cuiabá Brazil marianomphd@gmail.com; 2 Nurse, Master’s degree. Sentinel Surveillance Nurse in MS, Faculdade de Cuiabá - MT Brazil. Email: vitesinha@gmail.com. Corresponding author Faculdade Cuiabá Faculdade de Cuiabá Brazil vitesinha@gmail.com; 3 Nurse, Ph.D. Adjunct Professor III, Universidade do Estado de Mato Grosso (UNEMAT) Tangará da Serra - MT Brazil. Email: vagnernascimento@unemat.br Universidade do Estado de Mato Grosso Universidade do Estado de Mato Grosso Tangará da Serra Brazil vagnernascimento@unemat.br

**Keywords:** type 2 diabetes mellitus, glycated hemoglobin A, risk factors, control., diabetes mellitus tipo 2, hemoglobina A glucosilada, factores de riesgo, control., diabetes mellitus tipo 2, hemoglobina A glicada, fatores de risco, controle.

## Abstract

**Objective:**

To identify the proportion of poor of glycemic control and associated factors among people with type 2 diabetes attending a regional reference outpatient clinic in Mato Grosso (Brazil).

**Methods:**

This is a cross-sectional quantitative study based on data from medical records of 338 people with type 2 diabetes who attend a state reference outpatient clinic in Mato Grosso (Brazil). Information on glycemic control, sociodemographic factors, lifestyle and clinical conditions was collected.

**Results:**

The prevalence of elevated glycated hemoglobin was 47.34%. In the Poisson multiple regression model analysis with robust variance, poor glycemic control was significantly associated (p<0.05) with the following factors: insulin use (Prevalence Ratio -PR = 2.03), fasting glucose ≤70 and ≥100 mg/dL (PR = 2.0), postprandial glucose ≥180 mg/dL (PR = 1.76), no physical activity (PR = 1.62), the interaction between age group ≤59 years and the time of disease diagnosis >10 years (PR = 1.58), and presence of arterial hypertension (PR = 0.79).

**Conclusion:**

Most users of the reference outpatient clinic with type 2 diabetes had poor glycemic control associated with risk factors that alter glycated hemoglobin and negatively affect the achievement of established glycemic levels.

## Resumen

**Objetivo:**

Identificar la proporción de falta de control glucémico y los factores asociados entre las personas con diabetes tipo 2 que acuden a un ambulatorio regional de referencia en Mato Grosso (Brasil).

**Métodos:**

Se trata de un estudio transversal basado en los datos de las historias clínicas de 338 personas con diabetes tipo 2 atendidas en un ambulatorio estatal de referencia. Se tomó información del control glucémico, los factores sociodemográficos, el estilo de vida y las condiciones clínicas.

**Resultados:**

La prevalencia de hemoglobina glicosilada elevada fue del 47.34%. En el análisis del modelo de regresión múltiple de Poisson con varianza robusta mostró que el descontrol glucémico estaba significativamente asociado (p<0.05) con los siguientes factores: el uso de insulina (Ratio de Prevalencia -RP = 2.03), la glucemia en ayunas ≤70 y ≥100 mg/dL (PR = 2), la glucemia postprandial ≥180 mg/dL (PR = 1.76), la ausencia de actividad física (PR = 1.62), la interacción entre el grupo de edad ≤59 años y el tiempo de diagnóstico de la enfermedad >10 años (PR = 1.58) y la presencia de hipertensión (PR = 0.79).

**Conclusión:**

Una buena parte de los usuarios del ambulatorio de referencia con diabetes tipo 2 presentaba descontrol glucémico asociado a factores de riesgo que alteran la hemoglobina glicosilada y afectan negativamente la consecución de los niveles glucémicos establecidos.

## Resumo

**Objetivo:**

Identificar a proporção de falta de controle glicêmico e fatores associados entre pessoas com diabetes tipo 2 que visitam um ambulatório de referência regional em Mato Grosso (Brasil).

**Métodos:**

Trata-se de um estudo transversal e quantitativo, com base em dados de prontuários de 338 pessoas com diabetes tipo 2 atendidas em ambulatório de referência estadual de Mato Grosso (Brasil). Foi recolhida informação sobre controlo glicémico, factores socio-demográficos, estilo de vida e condições clínicas.

**Resultados:**

A prevalência de hemoglobina glicada elevada foi de 47.34%. Na análise do modelo de regressão múltipla de Poisson com variância robusta, foi demonstrado que o descontrole glicêmico foi significativamente associado (p<0.05) aos seguintes fatores: uso de insulina (Razão de Prevalência -RP = 2.03), glicemia de jejum ≤70 e ≥100 mg / dL (RP = 2.0), glicemia pós-prandial ≥180 mg / dL (RP = 1.76), nenhuma atividade física (RP = 1.62), a interação entre a faixa etária ≤59 anos e o tempo de diagnóstico da doença > 10 anos (RP = 1.58) e apresentar hipertensão arterial (RP = 0.79).

**Conclusão:**

Boa parte dos usuários do ambulatório de referência com diabetes do tipo 2 apresentou descontrole glicêmico associados a fatores de risco que alteram a hemoglobina glicada e afeta negativamente o alcance dos níveis glicêmicos estabelecidos.

## Introduction

Currently, about 463 million people have diabetes in the world, with estimates of reaching 700 million diabetic adults in 2045. This scenario also applies to Brazil, which ranks fifth among the ten countries with the highest number of diabetic adults, behind only China, India, the United States and Pakistan.(1) Specifically, type 2 diabetes mellitus (DM2), represents about 90% to 95% of all cases of diabetes worldwide, with higher proportions in low- and middle-income countries.(2) As DM2 is a chronic disease with disabling potential arising from its complications, it can have clinical, economic and social consequences for diabetic people, their families and the health system.(3) Type 2 diabetes mellitus can be the result of multiple factors, such as behavioral aspects (overweight, obesity, alcohol consumption, smoking), diet (refined carbohydrates and sugary foods, saturated fats), physical activity (physical inactivity, sedentary lifestyle), knowledge about one’s own health/disease condition, and self-care (non-adherence to health promotion and maintenance actions).(4) As a result, people with DM2 find it difficult to control in their daily lives, which requires trained professionals to assist them. In Brazil, a study conducted in 38 Basic Health Centers found that 69.8% of participants had uncontrolled DM2,(5) as in other countries.(1-6)

Some professions stand out in the care of diabetics. Nursing, for example, leads these care actions, from screening to self-management support and family management of diabetic people, identifying facilitators and barriers to diabetes control and resources to provide more effective care. Although nurses do not make the DM diagnosis on their own, without nursing care interventions, the adherence and maintenance of treatment and adoption of new essential lifestyles for glycemic control can make coping with the disease more difficult, costly and deleterious for everyone involved with the diabetic person. In this sense, the assessment and monitoring of glycemic levels of diabetic people are essential care actions performed by nursing, and have an important relationship with the reduction of morbidity (9.3%) and mortality (11.3%) rates globally.(1)

Nursing care for diabetics includes requesting tests for monitoring the disease and tracking complications. Among these tests, glycated hemoglobin (HbA1c) is considered the gold standard. This biomarker allows the estimation of glycemic values for a period of up to three months, diagnosis of the metabolic situation, provision of therapeutic guidance and assessment of the effectiveness of treatment in people with DM2.(7,8) According to the Brazilian Society of Diabetes,(9) HbA1c should be performed at least twice a year for individuals with favorable glycemic control and at least three times for those with difficulties in maintaining blood glucose levels under control. However, it is impossible to affirm that people with DM2 undergo this exam constantly. In the state of Mato Grosso, located in the Central West region of Brazil, among diabetics with a diagnosis time of more than 12 months, 40% have not had consultations in the last three months, impacting on blood glucose monitoring.(10) In the city of Cuiabá, capital of the state, 43% of diabetic patients at a Basic Health Center had HbA1c with values above the standardized target, a finding that indicates the vulnerability of this population to poor disease management.(11)

Difficulties in glycemic control are a problem of alarming proportions, which annually add extra burden to public health services and overload reference units. Therefore, the aim of this study was to identify the proportion of poor glycemic control and associated factors among people with type 2 diabetes who visit a regional reference outpatient clinic in Mato Grosso (Brazil).

## Methods

This is a cross-sectional, quantitative study conducted in a reference outpatient clinic in the state of Mato Grosso from November 2019 to March 2020. Mato Grosso is the third largest Brazilian state in terms of land area, the largest in the Center West region and part of the Amazônia Legal. The chosen outpatient clinic is the main one in the specialty of endocrinology in this state, as it receives and is the first reference center for the demands of 141 municipalities, with regular monitoring, free of charge by the National Health Service (Brazilian SUS). This clinic receives patients referred from other services, who can count on other specialists in the same space, such as nurses, social workers, pharmacists, nutritionists and psychologists, among others. There is also a laboratory for clinical analysis and the use of electronic medical records that allow the monitoring of this diabetic population.

This study included all patients over 18 years of age, with a DM2 medical diagnosis defined in their medical records and who presented a record of at least one laboratory test for Hb1Ac in 2019. For analysis of the HbA1c test, results from the last test performed in 2019 were considered. The study population was of the census type; 352 records were found at first, and after applying the inclusion and exclusion criteria, a total of 338 patients was reached. Data collection was performed in the Management Application system for University Hospitals - AGHU. Data were extracted digitally, organized in a spreadsheet with date of collection and subdivisions (demographic, laboratory “exams” and clinical diagnosis). Care was taken to include the medical record number in order to avoid duplicates. This process was conducted by one of the respondents, who was previously trained.

This system has several modules such as the Online Patient Record module and the outpatient care module. In the Online Patient Record, patient information is available electronically, clearly and with privacy control. This module provides access to registration data, clinical history, diagnoses and assistance provided (procedures, consultations, prescriptions, exams, guidelines and other activities of the patient in this clinic). Among the study variables, the dependent variable corresponded to glycemic control categorized according to the Brazilian Society of Diabetes.(9) Adult participants aged 18 to 59 years with HbA1c of less than 7% were considered to have adequate glycemic control and those with HbA1c greater than or equal to 7%, with inadequate glycemic control. In older adults over 60 years of age, HbA1c higher than 8.5% was considered inadequate control.

Independent variables included: (i) Sociodemographic aspects. Sex (male and female); age (18 to 59 years and 60 years or older); skin color (non-white and white); marital status (without a partner and with a partner); education (no schooling, less than or equal to 8 years, and more than 8 years); origin (other municipalities and municipality of Cuiabá); (ii) Lifestyle. Physical activity practice: no (when the individual performed physical activity less than twice or not once a week); no information (when the individual did not indicate if performed physical activity); and yes (when the individual performed physical activity at least twice a week);(12) and (iii) Clinical conditions. Postprandial blood glucose (greater than or equal to 180 mg/dL and less than 180 mg/dL) and fasting glucose (less than or equal to 70 mg/dL, greater than 100 mg/dL, and 70-100 mg/dL);(9) insulin use (yes and no); diagnosis time (greater than 10 years and less than or equal to 10 years); cardiovascular diseases: high risk (for those with a history of developing cardiovascular disease), cardiovascular disease (when the individual had a history of diagnosis of acute myocardial infarction, angina, heart failure) and not (for those with history of absence of risk); systemic arterial hypertension: yes (with history of diagnosis) and no (no history of diagnosis); amputation: yes (with history) and no (without history); obesity (yes and no) - measured based on the calculation of the body mass index (BMI) and classified as grade I, grade II, and grade III when BMI ≥ 30 kg/m; hospitalization: yes (admission for DM2 in the previous year) and no (no admission for DM2 in the previous year); retinopathy; neuropathy and nephropathy; ulcers; dyslipidemia; psychological (anxiety and depression). The latter were categorized into yes (with history of diagnosis) and no (no history of diagnosis). The clinical conditions of individuals were included in the medical records and identified by medical diagnosis.

Data were organized in Microsoft Excel version 2013 spreadsheets by double typing and later checked using the Data Compare tool. Then, the Statistical Package for the Social Sciences (SPSS) version 20 was imported for the analyzes. In the analysis of associations between the dependent variable and independent variables, a bivariate analysis was initially performed using the chi-square test and crude prevalence ratios with their respective 95% confidence intervals. After bivariate analysis, a multiple analysis using the Poisson multiple regression model with robust variance was considered. In this model, all independent variables that presented p<0.20 in the bivariate analysis were introduced using the stepwise forward process. In all inferences, significance levels less than or equal to 5% and 95% confidence were considered.

Data collection began only after approval by the Research Ethics Committee under CAAE: 22270519.2.0000.5541 and opinion number 3.675.333. An authorization term for the use of information from medical records was granted.

## Results

Among the study participants, most were females (73.08%), mean age of (58.07) years, standard deviation of 10.95. Almost half of all patients (47.34%) had uncontrolled blood glucose levels. Lack of glycemic control was also more prevalent among women (48.18%), non-white (47.62%), with less than eight years of schooling (43.46%), who lived with a partner (47.35%) and were from other cities (56.79 %). In Table 1, poor glycemic control was associated with the age of participants (p<0.001). In this case, altered HbA1c was 0.80 times more prevalent among those aged ≤ 59 years.

**Table 1:** Crude prevalence ratios of sociodemographic factors associated with elevated glycated hemoglobin test. Cuiabá, Mato Grosso, Brazil, 2020

Factors	Category	Glycated hemoglobin	Glycated hemoglobin	Glycated hemoglobin	Glycated hemoglobin	PR_c_	95% CI	***p*-value**
Factors	Category	Elevated	Elevated	Normal	Normal	PR_c_	95% CI	***p*-value**
Factors	Category	*n*	%	*n*	%	PR_c_	95% CI	***p*-value**
Sex(*n*=338)	Male	41	45.05	50	54.95	0.94	0.72 ; 1.21	0.610
	Female	119	48.18	128	51.82	1.00	-	-
								
**Age (*n*=338)**	≤59 years	97	62.18	59	37.82	1.80	1.42; 2.27	<0.001
	60 years or over	63	34.62	119	65.38	1.00	-	-
								
Skin color (N=332)	Non-white	150	47.62	165	52.38	1.16	0.65; 2.07	0.604
	White	7	41.18	10	58.82	1.00	-	-
								
**Marital status (*n*=324)**	No partner	45	45.92	53	54.08	0.97	0.75; 1.25	0.813
	With partner	107	47.35	119	52.65	1.00	-	-
								
**Years of schooling (*n*=327)**	No information	24	58.54	17	41.46	1.11	0.79; 1.56	0.554
	≤8 years	93	43.46	121	56.54	0.82	0.63; 1.08	0.170
	>8 years	38	52.78	34	47.22	1.00	-	-
								
**Origin (*n*=338)**	Other municipalities	46	56.79	35	43.21	1.28	1.01; 1.62	0.051
	Cuiabá	114	44.36	143	55.64	1.00	-	-

** PR_c_: crude prevalence ratio. 95% CI: 95% confidence interval. p: Significance level considering the chi-square distribution.

In Table 2, there is a statistically significant association between poor glycemic control with the categories: no physical activity, postprandial blood glucose ≥180 mg/dL, fasting blood glucose ≤70 and ≥100 mg/dL, yes for insulin use, diagnosis time >10 years, and high risk for cardiovascular disease.

**Table 2:** Crude prevalence ratios of lifestyle factors and clinical conditions associated with elevated glycated hemoglobin test. Cuiabá, Mato Grosso, Brazil, 2020

Category	Glycated hemoglobin	Glycated hemoglobin	Glycated hemoglobin	Glycated hemoglobin	PR_c_	95% IC	***p*-value**
Category	Elevated	Elevated	Normal	Normal	PR_c_	95% IC	***p*-value**
Category	*n*	%	*n*	%	PR_c_	95% IC	***p*-value**
Physical activity (*n*=315)							
No	118	56.46	91	43.5	1.87		<0.001
No information	10	43.48	13	56.5	1.44		0.218
Yes	32	30.19	74	69.8	1.00	-	-
Postprandial glucose (*n*=297)							
≥180 (mg/dL)	105	63.25	61	36.75	2.30		<0.001
<180 (mg/dL)	36	27.48	95	72.52	1.00	-	-
Fasting glucose (*n*=334)							
≤70 and ≥100 (mg/dL)	147	51.40	139	48.60	2.24		<0.001
70-100 (mg/dL)	11	22.92	37	77.08	1.00	-	-
Use of insulin (*n*=338)	116	62.70	69	37.3	2.18		<0.001
Diagnosis time (*n*=335)							
>10 years	72	55.38	58	44.6	1.29		0.026
≤10 years	88	42.93	117	57.1	1.00	-	-
Cardiovascular diseases (*n*=338)							
High risk	79	55.63	63	44.37	1.42		0.004
Diseases	11	61.11	7	38.89	1.55		0.074
No	70	39.33	108	60.67	1.00	-	-
Systemic arterial hypertension (*n*=338)	114	44.53	142	55.5	0.79		0.068
Amputation (*n*=338)	2	22.22	7	77.8	0.46		0.126*
Obesity (*n*=338)	72	52.17	66	47.8	1.19		0.139
Hospitalization (*n*=338)	6	75.00	2	25.00	1.61		0.156*
Retinopathy (*n*=338)	23	54.76	19	45.2	1.18		0.303
Neuropathy (*n*=338)	9	56.25	7	43.75	1.20		0.464
Nephropathy (*n*=338)	15	41.67	21	58.3	0.87		0.471
Ulcers (*n*=338)	13	54.17	11	45.8	1.16		0.487
Dyslipidemia (*n*=338)	81	48.21	87	51.8	1.04		0.748
Psychological anxiety and depression (*n*=338)	36	47.37	40	52.63	1.00		0.995

* PR_c_: crude prevalence ratio. 95% CI: 95% confidence interval. p: significance level considering the chi-square distribution. *: Fisher’s exact test.

In the analysis adjusted by the Poisson multiple regression model with robust variance, the categories that remained associated with high results of the glycated hemoglobin test were: insulin use, fasting blood glucose ≤70 and ≥100 mg/dL, postprandial blood glucose ≥180 mg/dL, no physical activity, interaction between age ≤59 years and time of disease diagnosis >10 years, origin, arterial hypertension (Table 3).

**Table 3:** Adjusted prevalence ratios for factors associated with elevated glycated hemoglobin test. Cuiabá, Mato Grosso, Brazil, 2020

Category	PR_a_	95% IC	***p*-value**
Insulin use			
Yes	2.03	(1.54; 2.69)	<0.001
No	1.00	-	-
Fasting glucose			
≤70 and ≥100 (mg/dL)	2.00	(1.23; 3.27)	0.006
De 70-100 (mg/dL)	1.00	-	-
Postprandial glucose			
≥180 (mg/dL)	1.76	(1.35; 2.29)	<0.001
<180 (mg/dL)	1.00	-	-
Physical activity			
No	1.62	(1.21; 2.16)	0.001
No information	1.06	(0.63; 1.79)	0.825
Yes	1.00	-	-
Interaction between age group and disease diagnosis time			
≤59 years and >10 years	1.58	(1.01; 2.46)	0.046
60 years or over and ≤10 years	1.00	-	-
Origin			
Other municipalities	1.41	(1.13; 1.76)	0.003
Cuiabá	1.00	-	-
Age group			
≤59 years	1.39	(1.05; 1.83)	0.021
60 years or over	1.00	-	-
Diagnosis time			
>10 years	0.76	(0.52; 1.11)	0.163
≤10 years	1.00	-	-
Systemic arterial hypertension			
Yes	0.79	(0.64; 0.98)	0.033
No	1.00	-	-

PR_a_: prevalence ratio adjusted by Poisson’s multiple regression model with robust variance. CI: confidence interval.

## Discussion

The prevalence of uncontrolled blood glucose levels in this study (47.34%) is lower than the values found in other studies in Brazil, such as in the South (69.08%)(5) and Southwest (70.2%) regions of Brazil,(6) where alterations in HbA1c were found, as well as in other parts of the world such as Western Ethiopia, where (59.05%) had poor glycemic control.(13) This lower prevalence among study participants is possibly related to the care and monitoring provided by this type of reference service, in which the therapeutic strategy is based on self-care practices for glycemic control. In addition, as this service integrates the university hospital facilities, educational activities and continuing professional training are developed with care guided by holistic, global and multidisciplinary care perspectives, in which creative processes of guidance, monitoring and intervention are tested, rethought and transformed into more viable therapeutic projects to maintain adequate blood glucose parameters and prevent complications.(14)

It is important that the reference service is used whenever the clinical conditions of diabetic patients indicate this need, without disregarding the referral/counter-referral integration. As diabetes is a sensitive condition in the primary care service, secondary prevention interventions should also be prioritized by it. Several factors that affect glycemic levels have a linear relationship with primary care services, namely repeating the medical prescription without reinforcing the guidelines of the therapy to be used, lack of management of indications for changes in lifestyle habits and even the little contact with patients who sporadically attend the health service.(15) However, given the large scope of primary care actions, some weaknesses faced in the service should be mentioned, such as the high turnover of health professionals, lack of adequate infrastructure, low technological density, weak support systems for diagnosis and monitoring of a chronic disease as diabetes.

The association between elevated HbA1c and the use of insulin was similar to that observed in other national(6-11) and international studies.(13-16) The use of insulin in this population is not effective in maintaining adequate glycemic levels, as DM2 management goes beyond drug therapy. Diabetics face barriers imposed by insulin therapy itself, such as repetitive injections and the respective discomfort, lack of supplies (materials) or access to home care, difficulty in maintaining regularity and adherence to therapy (food, medication and physical activity), as well as understanding the disease and its consequences that also interfere with glycemic control.(17) Still, it is noteworthy that the use of insulin revolutionized the treatment of the disease and the benefits arising from its regular, systematic and appropriate use provided better quality of life for diabetic patients.

The present study points to a significant association between HbA1c alterations with postprandial glucose and fasting glucose. Blood glucose outside its normal range affects the control of HbA1c and this finding was rarely observed in other studies. Undoubtedly, this is a differential that requires professionals’ greater perception of this fact and an appropriate management of the current condition, such as indication for fast acting insulin, if applicable,(18) or the use of other strategies, as mentioned in a Chinese study. It was found that a moderate intensity 20-minute walk after dinner can improve postprandial hyperglycemia, glycemic excursions and the normalization of fasting blood glucose in patients with DM2 with no potential risk of hypoglycemia.(19)

The association of elevated HbA1c with physical inactivity was also evidenced in a study conducted in Southwestern Bahia, Brazil.^4^) This association was expected, as evidence shows that inactive individuals or those with a low level of physical activity present worse glycemic levels.(20) Physical activity is one of the pillars in the treatment of chronic noncommunicable diseases, as it brings benefits in reducing mortality rates from cardiovascular diseases and preventing complications. Therefore, the defense and expansion of the new 2020 guideline on physical activity and sedentary behavior is recommended.(12)

However, it is essential to deepen the meaning of physical activity and health established between professionals and patients, based on health literacy that integrates levels of information processing for the decision-making process. Assessing how information is being understood and applied to maintain or improve health is crucial in the health-disease process. Diabetic individuals with low health literacy are more likely to have complications resulting from lack of glycemic control, because the fewer information resources the less adherence to health promotion actions.(21) Another important aspect is the patient’s individuality, as not all are equally able to perform physical activity regularly, even if of low intensity and without cost. Diabetic patients should be encouraged whenever possible and according to their ability, since reducing sedentary behavior is the best way to obtain positive health outcomes.(12)

Regarding age group, younger adults diagnosed with diabetes more than 10 years earlier were more likely to have poorer glycemic control compared to older patients with a relatively shorter diabetes diagnosis time (<10 years). Individuals aged 40 to 60 years with longer duration of the disease (>10 years) constitute a greater proportion of patients with poor glycemic control compared to those of different age groups.(16-22) This is a more recent reality, because although historically, poor glycemic control used to be more prevalent in older adults, nowadays, there is a higher incidence of diabetes in young people.(9) With the transformation of lifestyles and the approach to new care resources, there has been an increase in the longevity of this group and premature deaths of younger people resulting from exposure to risk factors (obesity, hypoactivity, alcoholism and smoking, another factor that can be attributed is the misuse of technologies in the contemporary world, added to the unavailability of time to perform physical activities, self-medication and others).(23) Hypertension was associated with poor glycemic control, a fact observed in another study.(5) Hypertension and DM2 are highly chronic diseases, and when developed jointly, they increase the risks of aggravation and deterioration of vital organs. In this context, one of the strategies to minimize such risks is based on the search for individuals who miss scheduled appointments, the survey of hospitalizations of hypertensive and diabetic patients in the community, and the activation of the formal and informal support network of these people, in order to monitor and strengthen the care bond.

In this study, the complexity of factors associated with the poor glycemic control identified, in addition to offering support for professional and scientific practice, reveals the diversity found in this care setting and the richness for the training of nurses experiencing this reality, as it is a field of development and continuous improvement of technical skills for students, teachers and health professionals. It is also an opportunity for nursing professors, tutors and preceptors to perceive and intervene in nursing students’ weaknesses in the monitoring of patients with this clinical profile, considered one of the greatest demands of Primary Health Care in Brazil. A recent study conducted in the United States (USA) highlighted that during the nursing consultation, older and more experienced students asked fewer questions to diabetic patients, but 32% were more empathetic and 76% offered more guidance than their younger colleagues. Such a scenario also brings a reflection on the levels and leveling of nursing students throughout their academic training for the care of these patients.(24) Identifying these particularities that interfere in the therapeutic care of diabetic patients can also signal failures in professional training, since the form and management of diseases, among other aspects, is related to the perception of severity and importance given by professionals.

Undoubtedly, nursing is equipped with knowledge and theories that guide and support health care, an essence that fosters interpersonal relationships and the creation of lasting bonds, and supports new lifestyles and the wellbeing of diabetic patients. In this context, thinking about the training of nurses in view of the prevalence of diabetics in the world, must include the premises of the profession, which acts not only after the confirmation of the clinical diagnosis, but in disease prevention and balance of human-nature relationships. In this direction, considering the advent of technologies strengthened during the COVID-19 pandemic, nurses and nursing students can empower themselves with telenursing as a resource for difficult-to-reach populations or those far from health services that enhances the effects of education in health and complements monitoring and surveillance measures. Furthermore, in recognizing the factors associated with poor glycemic control, this and other care technologies can improve the systematization of nursing care and consequently, predict and prevent the negative evolution of the disease.

## Conclusions

This study revealed that most users of the reference outpatient clinic with DM2 had poor glycemic control and factors associated with changes in HbA1c, namely younger adults, physically inactive, hypertensive, those using insulin, with changes in fasting and postprandial glucose tests, as well as the interaction between age group with longer disease diagnosis time. The findings indicated that diabetic individuals who do not keep blood glucose levels under control are vulnerable to a poor prognosis due to inadequate management. We highlight that health services, whether Primary Health Care or reference service for diabetics, must be circumspect in all conditions for the desirable reach of HbA1c in order to prevent complications, reduce morbidity and mortality and minimize the impact on public health. This requires the collective intervention on risk factors, and not only in the care setting, but in contexts of academic and professional training.

A limitation of the study was the lack of sociodemographic variables such as income, professional occupation and family history, which could help to better elucidate this scenario of poor glycemic control. However, for the first time in this Brazilian state, the variables associated with poor glycemic control in people with DM2 in this health service profile were identified. Given the importance of the findings, further research must be performed with a view to provide more evidence for the care of diabetics and consequently, reduce the number of referrals to reference services.
